# 同源结构域相互作用蛋白激酶2最新研究进展及其应用

**DOI:** 10.3779/j.issn.1009-3419.2011.04.13

**Published:** 2011-04-20

**Authors:** 文韫 贾, 宝兰 李, 文涛 岳

**Affiliations:** 1 101149 北京，北京市结核病胸部肿瘤研究所细胞室 Department of Cellular and Molecular Biology, Beijing TB and Toracic Tumor Research Institute, Beijing 101149, China; 2 101149 北京，北京胸科医院综合科 General Department, Beijing Chest Hospital, Bejing 101149, China

同源结构域相互作用蛋白激酶2（homeodomaininteracting protein kinase -2, HIPK 2）是近二十年来发现的一种定位在细胞核内，调节多种细胞、组织和器官功能的丝氨酸/苏氨酸激酶。HIPK 2是潜在的肿瘤抑制因子和DNA损伤相关激酶，它通过磷酸化反应作用于不同的下游靶点来激活凋亡进程。此外，HIPK 2还参与组织器官的形成、肿瘤的发生和发展。由于其作用广泛，近年来受到广大学者的关注。

## 1 HIPK 2的基本信息

上世纪90年代，Kim等^[1]^发现了一个由保守的蛋白激酶结构域和一个同源蛋白相互作用结构域组成的新的核蛋白激酶家族，由于该蛋白家族能够增强同源转录蛋白的活性，特命名为HIPK。目前HIPK家族包括3个成员：HIPK 1、HIPK 2和HIPK 3。*HIPK 2*基因包含13个外显子，大小约59×10^3^ bp，定位于7q33-7q34。*HIPK 2*高度保守，在小鼠定位于染色体6B。小鼠与人类之间HIPK 2氨基酸序列98%同源^[2]^。HIPK 2在各类组织中表达各异。我们在实验中也发现*HIPK 2* mRNA在各肺癌细胞系中表达量不尽相同。

HIPK 2含1, 189个氨基酸，属于DYRK激酶家族，作为同源蛋白的一个转录辅助抑制物具有多个功能域：①和同源蛋白相互作用的结合域；② PEST（proline glutamic acid serine or threonine）序列，是位于细胞内、半衰期不到2 h的富含脯氨酸、谷氨酸、丝氨酸及苏氨酸的区域，与蛋白的快速降解有关，因此哺乳动物细胞中很难获得该蛋白的信息^[3]^；③辅助抑制域；④位于C-端的YH序列；⑤位于N-端的蛋白激酶活化域。定位于细胞核内的蛋白质都具有的“核定位序列（nuclear localization sequence, NLS）”。

HIPK 2可以作用于多种功能性蛋白质，包括转录调节蛋白、染色质调节蛋白、细胞内信号转导蛋白、跨膜蛋白以及小泛素相关修饰连接酶（small ubiquitin-related modifer, SUMO）的E2成分^[4, 5]^。因此有学者^[6]^认为HIPK 2是重要的调节因子。

## 2 HIPK 2的功能

### 2.1 HIPK 2对靶点的调节

当细胞受到各种损伤时，HIPK 2会与肿瘤抑制因子p53形成复合物，磷酸化p53的Ser46，进而激活p53靶基因*puma*、*bax* 和*noxa* 等^[7-11]^。已经证实p53的Ser46在严重DNA损伤导致的凋亡中有重要作用^[12]^。HIPK 2还能磷酸化小鼠p53 Ser58（Ser46的同源位点）引发凋亡^[13]^。为了有效磷酸化p53，HIPK 2利用肿瘤抑制蛋白（promyelocytic leukemia, PML）作为辅助因子^[14]^。HIPK 2和PML将p53整合为PML-NBs^[15, 16]^而恢复p53活性^[17-19]^。HIPK 2还能与乙酰转移酶连接蛋白（CREB-binding protein, CBP）相互作用，在CBP帮助下，HIPK 2与p53定位于PML-NBs。在HIPK 2磷酸化p53 Ser46后，CBP乙酰化p53的Lys373和Lys382^[20]^。p53 Lys373/Lys382乙酰化在诱导凋亡和细胞老化中起着至关重要的作用^[11]^。

除了PML及其相关的NBs，Wnt/β-catenin信号通道的支架蛋白Axin^[21]^、PML-NB片段Sp100^[22]^、Sp70、Daxx^[23]^和p53诱导因子p53DINP1/TP53INP1^[24, 25]^都可以与p53和HIPK 2结合，作为HIPK 2磷酸化p53 Ser46的辅助因子^[26]^。例如：HIPK 2会以将β-catenin的Ser33和Ser37残基磷酸化和依赖蛋白酶体将β-catenin降解的方式，减弱Wnt/β-catenin信号通路，抑制体内外细胞增殖，但是当β-cateninatitsSer33和Ser37突变为丙氨酸时，HIPK 2将不能发挥上述作用^[27]^。

目前有发现在急性前髓细胞性白血病中HIPK 2时常与类维生素A受体-α（RARα）融合。由于DNA损伤，HIPK 2磷酸化PML的Ser8和Ser38。尽管HIPK 2介导的PML磷酸化只发生在DNA损伤反应的早期，但是具有致癌性的PML-RARα融合蛋白仍然受到强烈的迟发动力学效应，继续磷酸化；而且DNA损伤或者HIPK 2表达可以稳定PML和PML-RARα融合蛋白。N末端磷酸化部位导致DNA损伤引发的PML泛素化，而且它是PML与HIPK 2相互作用诱导细胞死亡的必需条件^[28]^。

另外，在几种上皮肿瘤中增强整合素α6和整合素β4的表达能促进肿瘤发展，但是具体机制不清楚。敲除HIPK 2能激活整合素β4转录，进而明显增加整合素β4依赖性促分裂原活化蛋白和AKT磷酸化。在p53表达的细胞（野生型wtp53细胞系）中保证HIPK 2水平就能抑制整合素β4的表达，缺失或者突变的p53细胞中却不会出现这种现象。这说明HIPK 2通过p53起作用。与体外实验相符的是表达p53的野生型人乳腺癌细胞中整合素β4过表达与HIPK 2活性受抑制有极其密切的关系。当消除HIPK 2和p53的功能后，p53家族的TAp63和TAp73能强烈激活整合素β4转录。总之，p53功能缺失有助于辅激酶蛋白与p53家族中TA成员结合，继而允许整合素β4转录^[29]^。

HIPK 2还表现出许多与p53无关的作用功能和方式。人类转录因子ZBTB4是HIPK 2的新靶点，两蛋白可以在体外相互作用，在体内也因局部化而相互结合。HIPK 2可以磷酸化ZBTB4的几个保守的残基，即795、797和983位苏氨酸。过表达HIPK 2可使ZBTB4降解，而过表达激酶缺陷型HIPK 2突变体则没有这种作用。HIPK 2的化学活性还能减少细胞中ZBTB4的数量，反之用药物或者RNA干扰可以增加ZBTB4水平。上述HIPK对ZBTB4负性调节都是在正常培养条件下进行的。另外，ZBTB4的降解也随着DNA损伤而增加。Yamada等^[30]^发现ZBTB4可以抑制p21转录、对抗损伤细胞的细胞周期停滞，引导其凋亡。因此HIPK 2对p21的活化可能有两条途径，刺激活化剂p53，同时抑制抑制剂ZBTB4。重要的是HIPK 2在作用过程中并没有磷酸化p53。

Rett综合症是一种神经系统的严重失常现象。甲基化的CG序列结合蛋白2（MeCP2）突变与Rett综合症和其它神经系统疾病有关，它通过增加各种辅阻遏物而抑制转录。最近发现，大脑中MeCP2发生Ser80、Ser229和Ser421磷酸化，调节MeCP2沉默活性。Bracaglia等首次鉴定出HIPK 2作为激酶可以在体内外与MeCP2结合并磷酸化其Ser80，而且HIPK 2必须与MeCP2的DNA结合才能磷酸化其Ser80，诱导凋亡。*HIPK 2*基因缺失小鼠的神经缺陷表明了其对正常大脑功能维持的重要作用^[31]^。

在p53缺失的细胞内，HIPK 2一样能够调控DNA损伤细胞的凋亡。HIPK 2将C末端结合蛋白CtBP Ser422磷酸化，使CtBP在蛋白酶体降解。而且，在p53缺失的肝癌细胞系内，p53通过激活JNK信号通路调节转化生长因子β（transforming growth factor-β, TGF-β）介导凋亡^[32]^。在紫外照射损伤时，JNK也能调节CtBP Ser422磷酸化及凋亡，这说明HIPK 2既能直接磷酸化CtBP，又能间接通过JNK协同取得相同的结果^[32, 33]^。

### 2.2 p53及其它因子对HIPK 2的调节作用

HIPK 2的功能反过来也会受到p53的调节，而且p53同时具有激活和抑制HIPK 2的能力。p53通过caspase途径作用于HIPK 2的916和977位天冬氨酸残基^[34, 35]^。更令人惊奇的是HIPK 2的生成过程中需要p53的靶基因caspase-6^[36]^。因此，p53可以利用其caspase通路反向调节HIPK 2的凋亡活性。

有资料^[37]^显示p53介导的E3泛素连接酶MDM2/HDM2可能是HIPK 2的负性调节因子，在DNA出现损伤时，它可以泛素化HIPK 2，并将其降解。

酵母双杂交试验证实WD40/SOCSbox蛋白（WSB）作为E3泛素连接酶，可以与HIPK 2相互作用。过表达WSB-1可以使其利用C末端降解HIPK 2。利用shRNA敲除内源WSB-1能增加内源HIPK 2的稳定性并使其聚集。在WSB-1中，主要是WD40重复序列和SOCS盒与HIPK 2结合并降解，而且WSB-1在体内外都能泛素化HIPK 2^[38]^。含保守序列WD-40蛋白的人类花色素苷调节剂基因11（Han11）可以干扰HIPK 2，体外Han11可直接与HIPK 2结合，通过HIPK 2调节激酶信号系统^[39]^。

## 3 HIPK 2与肺癌

p53对MDM2的负性调节非常敏感，MDM2通过自动调节通路可以明显抑制p53活性。由于应激，p53发生翻译后修饰，使其逃脱MDM2的控制，蓄积而激活。最近在肺癌细胞系中发现^[4]^，在MDM2参与下HIPK 2对p53有活化作用。DNA损伤后，HIPK 2连接并磷酸化p53，使其转录、凋亡，克服MDM2抑制。细胞发生DNA损伤后，可以通过阻止p53出核转运和MDM 2调节的蛋白化调节p53介导的凋亡。这就进一步阐释了HIPK 2/p53通路促进凋亡和抑制肿瘤形成的机制。

基因敲除小鼠胚胎成纤维细胞转录辅抑制子羧基末端凝结蛋白CtBP可以上调包括凋亡在内的多种基因。因此，有人预言可以通过调节细胞内CTBP的水平来调节凋亡能力。如前所述，HIPK 2就是这样一个因子，而且证实HIPK 2激活可以使CtBP Ser422磷酸化而降解。研究发现c-Jun氨基末端激酶1（c-Jun NH2-terminal kinase 1）活化也可以磷酸化CtBP Ser422并降解，使与p53无关的人肺癌细胞系凋亡。JNK1以前曾被认为与紫外线介导的凋亡有关，表达MKK7-JNK1或者紫外照射会通过蛋白酶体介导的通路减少CTBP水平。这一作用会因为JNK1缺失而受阻。此外，使用顺铂持续激活JNK1通路产生了类似的CTBP降解现象。这一结果为p53缺失型肿瘤提供了新的化疗靶点^[35]^。

当核酸毒性药物使DNA受损时，HIPK 2能使肿瘤抑制因子p53atSer46磷酸化，进而启动凋亡过程。HIPK 2在某些细胞内被以蛋白酶体依赖的和泛素连接酶Siah1部分依赖的方式泛素化降解而失活^[40]^。HIPK 2还是低氧诱导因子1α（hypoxia-inducible factor-1α, HIF-1α）的转录辅抑制子，可以抑制肿瘤血管生成和对抗耐药的产生。HIPK 2在肿瘤中由于缺氧等多种机制而摆脱调控。研究人员想通过恢复HIPK 2功能和抑制HIF-1α来研究缺氧问题，以此证实HIPK 2和p53与缺氧导致的耐药有关。因为增加HIF-1α表达或者以药物抑制p53功能就会损伤HIPK 2的功能，于是作者用低氧或者钴元素处理结肠和肺癌细胞系。钴能抑制HIPK 2对HIF-1α启动子的募集反应，低氧使p53靶点MDM2表达，并下调HIPK 2，当对MDM2进行RNA干扰后，恢复了用药时的HIPK 2/p53 Ser46磷酸化反应^[41]^。另外，虽然低氧可以不可逆的抑制HIPK 2，但是补充锌可以重新激活缺氧抑制的HIPK 2，使得HIF-1通路受到抑制，恢复p53 Ser46凋亡活性^[42]^。

## 4 HIPK 2的初步应用

如前所述，WSB-1对泛素化和降解HIPK 2有重要作用，但是当应用阿霉素和顺铂等DNA损伤药物时，该作用会被完全抑制。这就解释了一些肿瘤的耐药机制，并提供了解决问题的可能方向^[38]^。

鉴于HIPK 2/p53 Ser46通路的重要作用，有学者分别比较了顺铂敏感和抗药的卵巢癌细胞系2008和2008C13内药物引发的HIPK 2/p53 Ser46凋亡通路。药物（阿霉素和顺铂）不同，两细胞系内HIPK 2的表达也不同。在耐药细胞系内没检测到p53 Ser46凋亡通路。尽管2008C13细胞系耐受顺铂，但是对阿霉素介导的HIPK 2/p53 Ser46凋亡通路很敏感，而且敲除HIPK 2后，上述作用就会受到抑制。添加外源HIPK 2和转入p53 Ser46的靶基因AIP1能诱导耐铂细胞的凋亡。这些结果表明铂类耐药存在不同的药物激活通路调节HIPK 2和HIPK 2/p53 Ser46，外源性的HIPK 2可能成为治疗耐药野生型p53卵巢癌的新疗法^[43]^。

很长时间以来人们也在寻找无遗传毒性的特异性p53复活剂。MDM2对抗剂Nutlin-3和RITA是两种能够诱导有丝分裂停滞、临床学者所期盼的具有凋亡效应的药物。在经Nutlin-3处理的细胞系内，随着p53复活，MDM2将p53凋亡前活化剂HIPK 2降解，但是在RITA处理的细胞系内，短暂降低MDM2水平又会将HIPK 2激活。这表明MDM2对HIPK 2的调节对于决定细胞是有丝分裂停滞还是凋亡有很大作用，此外提示了欲利用MDM2对抗剂复活p53功能，可以将目光投在MDM2的靶点，而不是仅仅考虑p53本身^[44]^。

基于钴和低氧对HIPK 2的调节作用机理，在低氧环境细胞培养基中添加锌能增强

HIPK 2蛋白的稳定性和在细胞核内的聚集，使HIPK 2重新与HIF-1α启动子结合，抑制*MDR1*、*Bc12* 和*VEGF* 基因转录，激活p53凋亡对药物的反应。联合应用锌和ADR可以通过抑制HIF-1α通路和上调p53凋亡靶基因而明显抑制体内肿瘤生长^[41, 42]^。这对抗肿瘤新药的研发或者原有药品的改造和利用具有很好的提示作用。

由于使用阿霉素化疗所致的缺氧肿瘤细胞内，p53 Ser46磷酸化受损，肿瘤产生耐药，但是，应用蛋白酶体抑制剂能恢复缺氧肝细胞内HIPK 2的表达、p53 Ser46磷酸化和caspase活性。因此，蛋白酶体抑制剂可作为潜在方法，使缺氧肿瘤细胞对治疗仍然敏感^[40]^。

## 5 问题与展望

尽管目前人们对HIPK 2调节机制和信号通路有了更加深入的了解，但是仍有许多问题有待解决，如由于HIPK 2广泛的降调作用，人们普遍认为*HIPK 2*是一个肿瘤抑制基因，而且HIPK 2在乳癌、甲状腺癌以及血液学疾病中表达下调，但是在结肠癌和宫颈癌中表达比良性病变和正常组织中高^[45]^。这些差别的原因何在？低表达的原因有是否相同？是基于机制的不同，还是由于技术层面原因？在这一过程中是否出现了HIPK 2的突变，突变后作用如何？能否为相关肿瘤提供化学或基因治疗的现希望？这些问题都需要广大学者进一步深入研究。
